# The impact of submaximal fatiguing exercises on the ability to generate and sustain the maximal voluntary contraction

**DOI:** 10.3389/fphys.2022.970917

**Published:** 2022-09-02

**Authors:** Loïc Lebesque, Gil Scaglioni, Alain Martin

**Affiliations:** INSERM UMR1093-CAPS, Université Bourgogne Franche-Comté, UFR des Sciences du Sport, Dijon, France

**Keywords:** performance fatigability, maximal torque production, maximal torque sustainability, voluntary contraction, neuromuscular electrical stimulation, torque-time integral

## Abstract

Neuromuscular fatigability is a failure to produce or maintain a required torque, and commonly quantified with the decrease of maximal torque production during a few seconds-long maximal voluntary contraction (MVC). The literature shows that the MVC reduction after exercises with different torque-time integral (TTI), is often similar. However, it was shown that after a fatiguing exercise, the decline in the capacity to sustain the maximal voluntary contraction for 1 min (MVC_1-MIN_) differs from the decrease in the capacity to perform a brief-MVC, suggesting that this latter can only partially assess neuromuscular fatigability. This study aims to highlight the relevance of using a sustained MVC to further explore the neuromuscular alterations induced by fatiguing exercises with different TTI. We used two contraction intensities (i.e., 20% and 40% MVC) to modulate the TTI, and two exercise modalities [i.e., voluntary (VOL) and electrical induced (NMES)], since the letter are known to be more fatiguing for a given TTI. Thirteen subjects performed a plantar-flexors MVC_1-MIN_ before and after the fatiguing exercises. A similar MVC loss was obtained for the two exercise intensities despite a greater TTI at 40% MVC, regardless of the contraction modality. On the other hand, the torque loss during MVC_1-MIN_ was significantly greater after the 40% compared to 20% MVC exercise. These findings are crucial because they demonstrate that maximal torque production and sustainability are two complementary features of neuromuscular fatigability. Hence, MVC_1-MIN_ assessing simultaneously both capacities is essential to provide a more detailed description of neuromuscular fatigability.

## Introduction

Performance fatigability is commonly defined as an objective decline in motor performance (Kluger et al., 2013) such as a failure to produce or maintain the required torque (Edwards, 1981). This phenomenon develops progressively from the onset to the exercise end, and it involves muscular changes occurring within the muscle as well as a failure of the central nervous system to adequately drive the motoneurons (Gandevia, 2001). In accordance with the previous definition, the protocol classically used to quantify fatigability requires recording of maximal torque during a brief (i.e., a few seconds long) maximal voluntary contraction (MVC), performed before and after a fatiguing exercise. Additionally, percutaneous stimulations of the motor nerve are generally used to estimate the relative contributions of neural and muscular processes to the decline in MVC (Millet et al., 2011).

The decline in maximal torque production (i.e., MVC loss) has been widely used in the literature to assess the neuromuscular fatigability induced by ecological exercises, like running or cycling, but also by isometric exercises involving a specific muscle group or task. For instance, some studies investigated the impact of isometric exercises carried out with different muscle activation modalities on maximal torque production. Muthalib et al. (2010) observed that the same number of elbow flexors contractions, evoked by neuromuscular electrical stimulations (NMES) or voluntarily performed (VOL), induced the same MVC loss, but for a muscle torque-time integral (i.e., the total amount of torque produced during the exercise, TTI) that was twice lower in NMES. Similarly, Hachard et al. (2019) compared the MVC loss induced by VOL and NMES isometric contractions of knee extensors at 20% MVC, performed until task failure. The authors observed that the MVC loss of the knee extensors was identical for these two exercise modalities although the time to task failure, and thus the TTI, were greater in VOL. Taken together, these results suggest that VOL and NMES exercises are equally altering the maximal torque production capacity, despite a different TTI. It, therefore, appears that the MVC loss caused by these exercises is independent from TTI. This consideration led us to look for an index sensitive to the exercise TTI.

To explore neuromuscular fatigability, Byrne and Eston (2002) used, in addition to the brief MVC, a MVC sustained for 1 min (MVC_1-MIN_) before and after an eccentric exercise. These authors showed that 10 × 10 eccentric contractions of the quadriceps muscle, performed at 80% of 1-repetition maximum, differently affects maximal torque production capacity (i.e., torque loss during a brief MVC), and maximal torque sustainability (i.e., torque loss during a MVC_1-MIN_); the former was reduced while the latter was not. This result suggests that loss of maximal torque production and maximal torque sustainability represent distinct aspects of neuromuscular fatigability. The MVC loss provides an insight into nervous and/or muscular alterations induced by muscle exercise (Boyas and Guével, 2011; Kent-Braun et al., 2012). Whereas, torque loss during a sustained MVC reflects the adaptive response of the neuromuscular system to maintain a high level of torque despite the nervous and/or muscular alterations induced by exercise.

We thought to consider the torque decline observed during a prolonged MVC, because a protracted contraction could be more affected by the energy expenditure generated by a fatiguing exercise (assessed by the TTI) and other homeostatic parameters (Boska et al., 1990; Davis and Bailey, 1997; Gandevia, 1998; Kent-Braun, 1999; Amann et al., 2013), than a short effort (brief-MVC). Because a MVC_1-MIN_ can assess both maximal torque production and sustainability, this study aims to highlight the relevance of using a sustained MVC to further explore the neuromuscular alterations induced by fatiguing exercises with different TTI. The relevance of this study lies in the fact that it could emphasize the necessity of assessing both capacities when aiming to characterize neuromuscular fatigability. To this end, we proposed to use a sustained MVC for 1 min (MVC_1-MIN_) before and after submaximal fatiguing exercises with different contraction modalities (VOL and NMES) and muscle TTIs (contractions at 20% and 40% MVC). NMES contractions are known to be more fatiguing than VOL ones, when muscle TTIs are matched, as indicated by the greater MVC loss induced by muscle exogenous activation (Theurel et al., 2007; Jubeau et al., 2008). Thus, the comparison between these two contraction modalities, at two levels of muscle TTI, seems to be the adequate experimental paradigm to evaluate the interest of associating indexes of maximal torque production and sustainability since the former will be more impacted by the contraction modality (NMES vs. VOL) and the latter by the muscle contraction level (20% vs. 40% MVC). Our hypothesis is that the fatiguing exercise with the greatest TTI induces the greatest loss of torque during the sustained MVC (∆MVC_1-MIN_). Finally, because reductions in maximal torque production and sustainability are transient phenomena, our second aim was to investigate the recovery of MVC loss and ∆MVC_1-MIN_ after a 10-min rest period.

## Materials and methods

### Subjects

Thirteen healthy volunteers (three females; age: 23.5 ± 2.0 years, body mass: 72.2 ± 15.7 kg, height: 177.6 ± 9.2 cm, body mass index: 22.7 ± 3.2 kg m^−2^) without neurological or physical disorders gave written informed consent to participate in this study. All subjects were right-footed and physically active [Physical Activity Scale (Robert et al., 2004): 21.5 ± 3.9]. The protocol was approved by the CPP SOOM III ethics committee (number 2017-A00064-49; ClinicalTrials.gov Identifier: NCT03334526) and complied with the Declaration of Helsinki.

### Protocol

#### Experimental design

All subjects took part in four experimental sessions randomly administered. The sessions lasted 1.5 h each and were interspaced by an interval of 7 days. For each subject, sessions were planned at the same time of the day. Each session included a neuromuscular assessment of plantar flexors (PF) (torque and electromyographic (EMG) recordings) before (Pre) and after a submaximal fatiguing exercise (Post), and after a recovery period of 10 min (Post-10). The submaximal fatiguing exercise started 3 min after the completion of the pre-exercise neuromuscular assessments. This fatiguing exercise changed among sessions. Briefly, it consisted of neuromuscular electrical stimulation (NMES) or voluntary contraction (VOL) performed each at two submaximal intensities (i.e., 20% and 40% MVC). Six out of thirteen subjects also participated in a control session during which only pre and post-tests were performed without fatiguing exercise.

#### Neuromuscular assessment

The neuromuscular assessment was preceded by a standardized warm-up protocol for PF. Subjects were asked to perform five isometric contractions at 25% of their perceived maximal effort, 3% at 50%, and 2% at 80%, each lasting 5 s. Two MVC lasting 5 s with 30-s rest in-between were performed to ensure that subjects were ready to start the neuromuscular assessment. Then, a 1 min-rest period was given to the subjects before starting the neuromuscular assessment. The neuromuscular assessment consisted in a MVC_1-MIN_ of PF. A single percutaneous stimulation of the tibial nerve at rest gave subjects the signal to start the sustained contraction. Electrical doublets superimposed to the contraction were delivered at the same time during the MVC_1-MIN_ (i.e., 5 and 55 s after the single stimulation). To ensure maximal performance, loud verbal encouragements were given to the subject during the effort. In addition, participants were informed of the MVC_1-MIN_ duration to lessen the adoption of a pacing strategy (Halperin et al., 2014) during the sustained contraction. The choice to set to 1 min the duration of the sustained MVC was made because it has been shown that the greatest torque loss occurs during the first minute of contraction (Gandevia et al., 1996; Kent-Braun, 1999; Post et al., 2009).

#### Fatiguing exercise

The fatiguing exercise was composed of intermittent isometric contractions of PF (10 s on/5 s off, duty cycle = 67%). In VOL session, subjects had to adjust their torque to a visual target-torque feedback displayed on a computer screen placed in front of them (according sound signals to respect the duty cycle), while in NMES session, the stimulation intensity was set to reach the target torque and kept constant. The TTI produced during each fatiguing exercise was controlled with a custom-made system made of a voltmeter (5XP-A, Amprobe) and synchronized with the dynamometer. In the first session of each exercise intensity (20% and 40% MVC), the number of fatiguing contractions was set to 40, regardless of the exercise modality (NMES or VOL). In the second session at the same intensity, but performed in the remaining modality, the number of contractions was adjusted to obtain a similar TTI in both modalities.

To ensure that pre-exercise MVC_1-MIN_ did not affect post-test torque production, six out of 13 subjects had an extra session without the fatiguing exercise, but with a 10-min rest period (corresponding to the time of exercise) between the neuromuscular assessments (i.e., control session).

**FIGURE 1:**
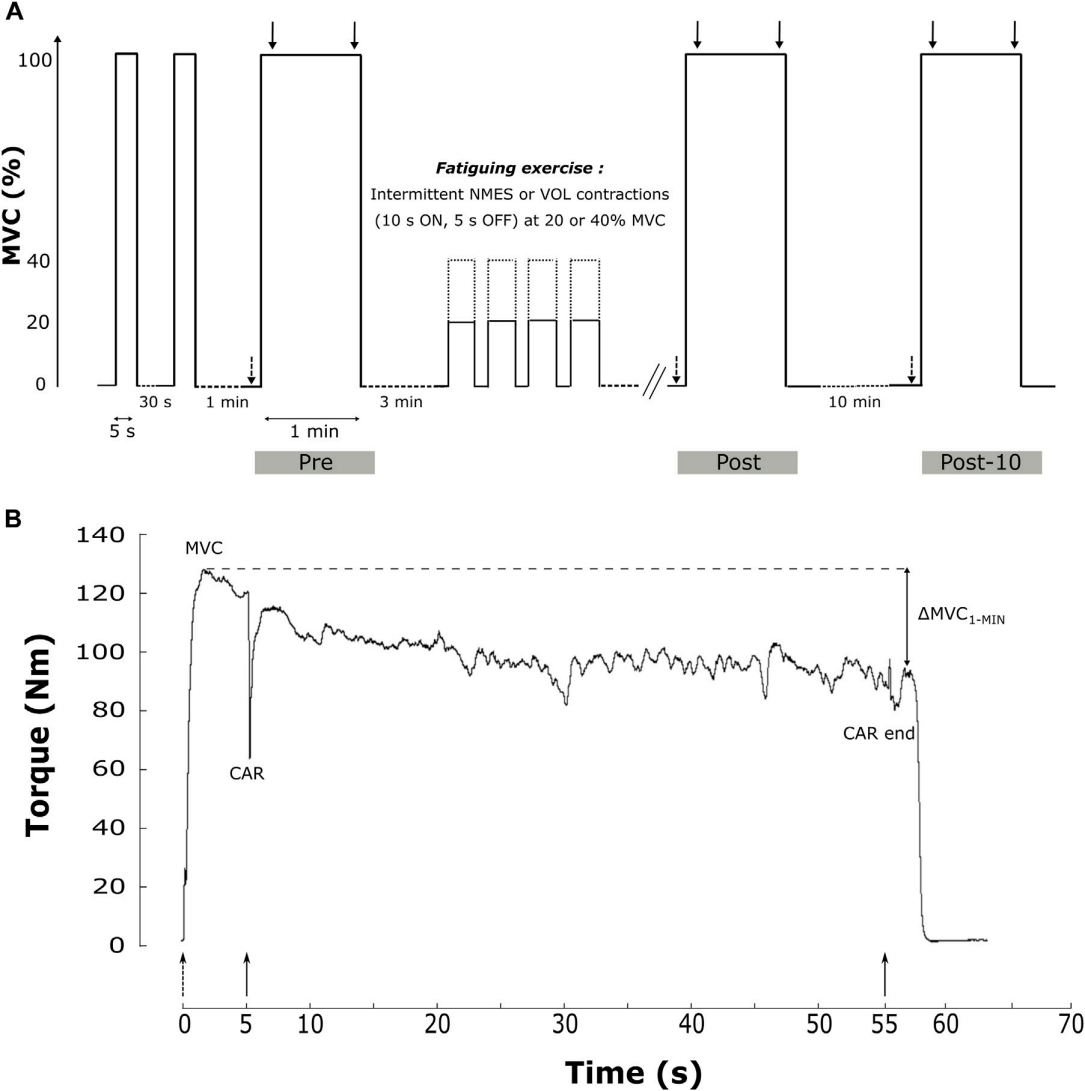
Sum up of the experimental design of the study. **(A).** The protocol included two brief-MVC and a 1 min sustained MVC (MVC_1-MIN_) before (Pre), after (Post) the fatiguing exercise and after a 10 min rest period (Post-10). The fatiguing exercise was an intermittent task with 10 s of contraction and 5 s of rest, in VOL or NMES modalities and at 20% (solid line) or 40% MVC (dashed line). Single (dashed arrow) and 100 Hz doublet (solid arrow) electrical stimulations are delivered during the protocol. **(B).** Data of torque production during the MVC_1-MIN_ from one representative subject before the fatiguing exercise. Main studied parameters (MVC, CAR and ∆MVC_1-MIN_) are reported. Dotted up arrows represent single electrical stimulations of PF at rest before the MVC_1-MIN_, while solid down arrows represent double electrical stimulations (100 Hz) superimposed at the start and the end of the MVC_1-MIN_. CAR = central activation ratio; MVC = maximal voluntary contraction; MVC_1-MIN_ = 1-min sustained MVC; ∆MVC_1-MIN_ = torque loss during the sustained MVC; VOL = voluntary fatiguing exercise; NMES = neuromuscular electrical stimulation fatiguing exercise; 20% and 40% MVC represent the intensity of the fatiguing exercise.

The experimental protocol is depicted in Figure 1.

### Data acquisition

#### Torque recordings

Voluntary and electrically-evoked PF isometric torque was recorded using an isokinetic dynamometer (Biodex Medical System Inc. Shirley, NY, United States). Subjects were comfortably seated on the chair of the dynamometer with the right foot securely strapped to the footplate at the ankle level. The centre of rotation of the dynamometer shaft was aligned with the lateral malleolus. The ankle angle was set at 90°, the knee joint was at 110° and the hip angle was at 130° (180° full extension). To limit trunk movements that could affect torque development, the hip was firmly strapped to the seat with a belt. During fatiguing exercises, subjects had to adjust their torque to a visual feedback of the target torque displayed on a computer screen placed at 2 m in front of them for VOL sessions while the stimulation intensity is initially set to reach the target torque during NMES and kept constant. The setting configuration was kept constant throughout the entire experimental session and among sessions.

#### EMG recordings

Electromyographic activity was recorded from four muscles of the right leg: the soleus (SOL), the gastrocnemii medialis (GM) and lateralis (GL), and the tibialis anterior (TA). EMG recording of the antagonist muscle allowed checking that no inadvertent activation of this muscle occurs during tibial nerve stimulation. EMG was recorded bipolarly using silver chloride circular surface electrodes (7 mm recording diameter) (Contrôle Graphique Medical, Brie-Compte-Robert, France) with an inter-electrode distance (centre-to-centre) of 20 mm. To minimize skin impedance (<5 kΩ), the skin was shaved, abraded and cleaned with alcohol, before electrode positioning. For SOL, electrodes were positioned 3 cm below the insertion of the two gastrocnemii over the Achilles’ tendon. For GM and GL, electrodes were placed over the muscle bellies. For TA, they were positioned on the upper third of the distance between the fibula head and the tip of the lateral malleolus. The reference electrode was placed between the two gastrocnemii bellies of the same leg. EMG signals were amplified (gain = 1,000) and filtered (10 Hz–500 Hz). Torque and EMG data were recorded at a sampling frequency of 2 kHz with the Biopac acquisition system (Biopac System Inc., United States) and stored with commercially available software (Acqknowledge MP150) for off-line analysis.

### Stimulation

#### Nerve stimulation test

Percutaneous electrical stimulation of the posterior tibial nerve in the popliteal fossa was carried out to evoke electrophysiological and mechanical responses of the triceps surae muscles. Monophasic rectangular pulses (1 ms) were delivered using a Digitimer stimulator (DS7AH, Digitimer, Hertfordshire, United Kingdom), triggered by a commercially available software (Tida, Heka Elektronik, Lambrecht/Pfalz, Germany). The anode (5 cm × 10 cm, Compex SA, Ecublens, Switzerland) was placed beneath the patella. The optimal stimulation site, namely the site where the greatest M-wave amplitude in SOL was evoked, was located with a hand-held cathode ball (0.5 cm diameter). Once the stimulation site was determined, the cathode (a self-adhesive electrode with 7 mm diameter, Contrôle Graphique Medical, Brie-Compte-Robert, France) was firmly fixed to this site with tape. The intensity of stimulation was progressively increased by 5 mA from the M-wave threshold to the maximal M-wave (M_MAX_). The intensity at which M_MAX_ amplitude plateaued (i.e., no further increase in the M-wave amplitude was evident in SOL) was further increased by 20% to ensure a supramaximal stimulation. This stimulation intensity was kept constant throughout the experimental session (mean supramaximal intensity was 65.3 ± 30.3 mA). Electrical doublets superimposed to the contraction during the MVC_1-MIN_ were delivered at supramaximal stimulation intensity with an inter-pulse duration of 10 ms (100 Hz).

#### Electrically evoked fatiguing exercise

PF NMES contractions were evoked using two rectangular electrodes (5 cm × 10 cm, Compex SA, Ecublens, Switzerland) placed over the triceps surae muscle, respectively 5 cm beneath the popliteal fossa and on the insertion point of the two gastrocnemii muscles to the Achilles tendon, around 2 cm above SOL EMG electrodes. The NMES fatiguing intermittent exercise consisted of trains of monophasic rectangular pulses (50 Hz, 500 µs) lasting 10 s interspaced by 5 s and delivered to PF via the Digitimer stimulator. The stimulation intensity was progressively increased in order to achieve 20% or 40% MVC, according to the experimental session, and kept constant during the NMES protocol.

### Data analysis

#### Torque analysis

MVC was determined as the highest torque achieved during the first 5 s of the MVC_1-MIN_. To ensure that participants did not adopt a pacing strategy that could influence the MVC measure (Šambaher et al., 2016), we also measured the maximal torque reached during brief-MVC to compare it with pre-exercise MVC. The pre-exercise MVC was used to set the target torque of the fatiguing exercise. The MVC loss corresponds to the relative difference between the MVC values recorded before and after the fatiguing exercise. The torque loss during the MVC_1-MIN_ (∆MVC_1-MIN_) was calculated as the relative change between the MVC value and the mean torque of the last 5 s of contraction as follows:

$$\Delta MVC_{1-MIN}=\;\left[\left(\frac{final\;torque}{MVC}\right)-1\right]\times 100$$ (1)

Change in ∆MVC_1-MIN_ between pre and post-exercise was quantified as the difference between both measures:

$$\Delta MVC1-MIN\;change\;=\;Pre-exercise\;\Delta MVC_{1-MIN}\;\;-\;Post-exercise\;\Delta MVC_{1-MIN}\;$$ (2)

The central activation ratio (CAR) was calculated at the beginning and the end of the MVC_1-MIN_ (after 5 and 55 s of contraction, respectively), according to the formula of Gandevia et al. (1996):

$$CAR\;=\;\left[1-\left(\frac{superimposed\;twitch}{MVC+surimposed\;twitch}\right)\right]\times 100$$ (3)

A value of 100 indicate a full central activation. The ratio between the CAR at the end and at the beginning of the MVC_1-MIN_, expressed in percentage of change, is called ∆CAR_1-MIN_ and represents the evolution of central activation during the MVC_1-MIN._ The ∆CAR_1-MIN_ was calculated with the following equation:

$$\Delta CAR_{1-MIN}=\;\left[\left(\frac{CAR\;end}{CAR\;beginning}\right)-1\right]\times 100$$ (4)

The TTI of the fatiguing exercise represents the total amount of torque produced by the PF and corresponds to the integral of the entire exercise period. This time window started at the onset of the raising phase of the first fatiguing contraction and ended after the decline phase of the last one.

#### EMG analysis

Considering that SOL, MG and LG concomitantly contribute to the plantar flexion mechanical response, we chose to express the EMG data (EMG activity and M_MAX_) as the sum of these three triceps surae muscles. The sum of the peak-to-peak amplitude of M_MAX_ of the three triceps surae muscles, evoked at rest, before and after each MVC_1-MIN_, was calculated (ΣM_MAX_). The EMG activity of each muscle during the MVC_1-MIN_ was quantified by the root mean square (RMS) value of the raw EMG signal. The RMS associated with the peak force was calculated from 250 ms prior the MVC to 250 ms after for each muscle and summed (ΣRMS_MVC_). This sum was then normalized with respect to the ΣM_MAX_ recorded before the MVC_1-MIN_ (ΣRMS_MVC_/ΣM_MAX_). The ΣRMS value was also calculated 500 ms prior to the second superimposed doublet (i.e., 55 s after the MVC_1-MIN_ start) and normalized by the ΣM_MAX_ recorded, at rest, after the MVC_1-MIN._ Then, this latter ratio was expressed as the relative change with respect to ΣRMS_MVC_/ΣM_MAX_ (∆RMS/ΣM_MAX_) to evaluate muscle activity changes during the MVC_1-MIN_.

#### Statistical analysis

Normality criteria were tested using the Shapiro-Wilk test. When the normality test failed, non-parametric analysis was performed. The factors used for ANOVA analysis were condition (NMES and VOL), intensity (20 and 40% MVC), time (pre, post and post-10) and *measure* (brief and sustained contraction). Paired t-tests were performed on MVC and ∆MVC_1-MIN_ obtained during the control session (pre vs. post). Two-way factorial ANOVA (condition and intensity) on fatiguing exercise TTI was conducted. Three-way ANOVAs with repeated measures (condition, intensity, and time) were performed on MVC, MVC loss, and ∆MVC_1-MIN_. A Three-way ANOVA with repeated measures (*condition*, *intensity* and *measure*) was performed on MVC before the fatiguing exercise. When a significant main effect or interaction was found, a post-hoc analysis was made using the HSD Tukey’s test. The Non-parametric related samples Friedman’s two-way ANOVA by rank with condition and intensity factors was used for contraction number during the fatiguing exercise. Friedman’s three-way ANOVAs with condition, intensity, and time factors were conducted on CAR and ∆CAR_1-MIN_, ΣRMS_MVC_/ΣM_MAX_ and ∆RMS/ΣM_MAX_, and on ΣM_MAX_. When the Friedman’s ANOVA revealed an effect, the Wilcoxon’s signed-rank test for paired multiple comparisons (with a Bonferroni correction) was performed. Correlations between exercise TTI and torque loss variables (MVC loss and ∆MVC_1-MIN_ change) were analysed using repeated measures correlations (rmcorr R package, Bakdash and Marusich, 2017). We calculated the intraclass correlation coefficient (ICC) for the maximal torque from brief MVC and MVC_1-MIN_ before the fatiguing exercise. The ICC calculation was based on a mean-rating (k = 2), absolute-agreement, 2-way mixed-effects model estimates and their 95% confident intervals (CI_95%_). Values less than 0.5 are indicative of poor reliability, values between 0.5 and 0.75 indicate moderate reliability, values between 0.75 and 0.9 indicate good reliability, and values greater than 0.90 indicate excellent reliability (Koo and Li, 2016).

It was estimated that thirteen participants were needed to detect differences with moderate effect size (η_p_^2^ = 0.08) using standard parameters of 1−β = 0.95 and α = 0.05. The critical level for statistical significance was set at 5%. Effect sizes of parametric ANOVA are reported as partial eta squared (η_p_^2^) with small (≥0.01), moderate (≥0.07) and large effects (≥0.14). Effect sizes (ES) were also calculated for parametric (Cohen’s d) and non-parametric comparisons (Cohen’s r) (Fritz et al., 2012). Small, moderate and large effects are considered for Cohen’s d ≥ 0.2, ≥0.5, ≥0.8, and Cohen’s r ≥ 0.1, ≥0.3, ≥0.5 respectively. Results are reported as mean ± standard deviation. Repeated measures correlations were performed and plotted with R (version 4.1.3, R Core Team, 2017). ICC analysis was performed with SPSS Statistical package (SPSS Inc., version 22, Chicago, IL, United States). Other frequentist statistical tests were performed using Statistica software (Statsoft, version 12, Tulsa, OK, United States) except for power analysis and the Cohen’s effect size that were calculated with G*Power software (version 3.1.9.2, Universität Düsseldorf, Germany).

## Results

### Control session

In subjects who performed the extra session without fatiguing exercise, no significant difference was observed between pre and post-test for MVC (120.5 ± 15.7 Nm vs. 121.7 ± 11.9 Nm respectively, t (5) = −0.54, *p* = 0.613, CI_95%_ = [−6.736: 4.403], ES = 0.086) and ∆MVC_1-MIN_ (−31.1% ± 5.2% vs. −29.8% ± 4.6% respectively, t (5) = −1.60, *p* = 0.171, CI_95%_ = [-3.346: 0.779], ES = 0.265). Thus, it may be concluded that the pre-test MVC_1-MIN_ did not affect the post-test MVC and ∆MVC_1-MIN_, analysed after a 10 min rest period (corresponding to the time of exercise).

#### Fatiguing exercise

As the TTI of the fatiguing exercises was matched for the same intensity between modalities, the statistical analysis only revealed a significant *intensity* effect (F_1,12_ = 94.802, *p* < 0.001, η_p_^2^ = 0.888). As expected, the TTI was higher in 40% than in 20% MVC intensity (20,438 ± 2,939 Nms vs. 12,009 ± 2,340 Nms, *p* < 0.001, ES = 3.173). Because torque production during NMES contractions was not as steady as in the VOL condition, we let participants to perform more contraction in NMES condition than in VOL condition, for both contraction intensities (20% MVC: NMES = 51 ± 16 vs. VOL = 35 ± 6, Z = 3.06, *p* = 0.002, ES = 0.849; 40% MVC: NMES = 50 ± 11 vs. VOL = 37 ± 4, Z = 3.18, *p* = 0.002, ES = 0.882).

### Assessments related to maximal torque production capacity

#### Torque

To ensure that MVC_1-MIN_ provide a reliable index of maximal torque production, the maximal torque recorded during the brief and the MVC_1-MIN_ were compared before the fatiguing exercise. No significant difference between these values was detected (F_1,12_ = 0.818, *p* = 0.384, η_p_^2^ = 0.064). In addition, the ICC of MVC between both measures was 0.951 (CI_95%_ = 0.915–0.972), indicating an excellent reliability.

**FIGURE 2:**
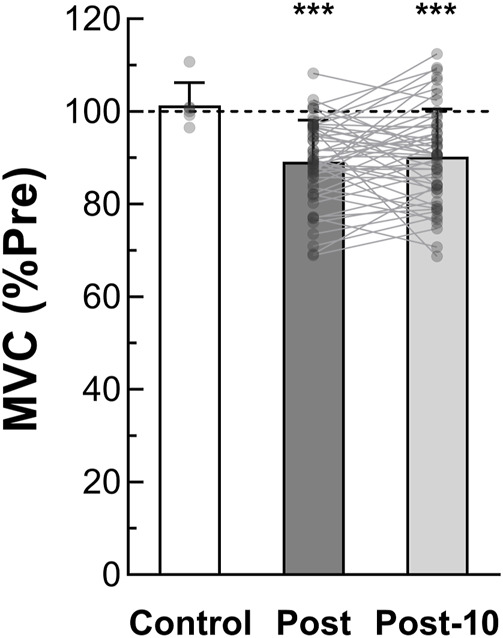
Maximal voluntary torque production (*n* = 13). MVC is determined as the maximal voluntary torque generated during the MVC_1-MIN_. Pooled Post-exercise and Post 10-min recovery period MVC are expressed as a percentage of pre-exercise value. MVC after the control session (without fatiguing exercise, *n* = 6) is also reported. *** Significant difference from pre-exercise (*p* < 0.001). MVC = maximal voluntary contraction; MVC_1-MIN_ = 1-min sustained MVC; Pre = before the fatiguing exercise; Post = immediately after the fatiguing exercise; Post-10 = after the 10-min rest period following the fatiguing exercise.

**FIGURE 3:**
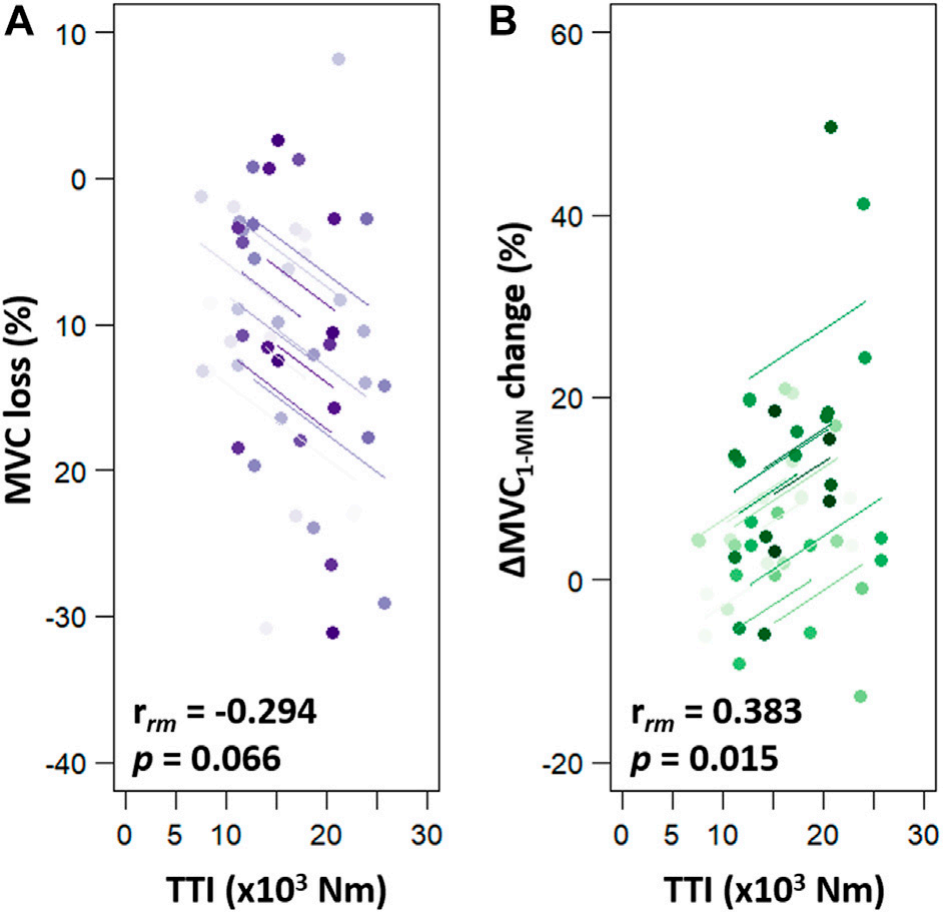
Repeated measures correlations (rmcorr) between exercise TTI and torque production changes (MVC loss, panel **(A)**; ∆MVC_1-MIN_ change, panel **(B)**. MVC loss corresponds to the percent of MVC loss from before to after the fatiguing exercise. ∆MVC_1-MIN_ change corresponds to the difference in ∆MVC_1-MIN_ between before and after the fatiguing exercise. In each panel, four similar colour dots represent each participant’s data from each session sessions and coloured lines show rmcorr fits for each participant. MVC = maximal voluntary contraction; ∆MVC_1-MIN_ = torque loss during a 1-min sustained MVC; TTI = exercise torque-time integral.

By comparing MVC among protocol time points, we observed a significant *time* effect (F_2,24_ = 35.503, *p* < 0.001, η_p_^2^ = 0.747) whereas no *condition*, *intensity* or interaction effect was found. As depicted in Figure 2, pooled pre-exercise value (110.6 ± 23.5 Nm) was significantly higher compared to post-exercise (98.2 ± 21.3 Nm, *p* < 0.001) and post-10 min (99.3 ± 21.7 Nm, *p* < 0.001). No significant difference in MVC between post and post-10 was observed (*p* = 0.769). Moreover, no significant correlation was detected between post-exercise MVC loss and exercise TTI [r_rm_ (38) = −0.294, *p* = 0.066, CI_95%_ = (−0.561 : 0.029)] (Figure 3A).

#### CAR and RMS

Before the fatiguing exercise, the CAR was similar among sessions (Table 1; Z-range: 0.314–0.734; all *p* = 1.000; ES-range: 0.087–0.204). The only exercise that had an impact on the CAR was the VOL 40% MVC (−3.5% ± 4.0%, Z = 3.18, *p* = 0.005, ES = 0.882). After the recovery period, the CAR was slightly lower, compared to the CAR calculated before the fatiguing exercise, for all sessions (EMS 20% MVC: −1.0% ± 1.1%, Z = 2.970, *p* = 0.009, ES = 0.824; VOL 20% MVC: −1.2% ± 1.2%, Z = 2.481, *p* = 0.039, ES = 0.688; EMS 40% MVC: −1.2% ± 0.9%, Z = 3.180, *p* = 0.004, ES = 0.882), except for the VOL 40% MVC (Z = 2.062, *p* = 0.118, ES = 0.572). For this latter session, the CAR seems to have completely recovered following the recovery period, even though the post-10 value was not different from the post exercise (Z = 1.49, *p* = 0.408, ES = 0.413) (Table 1).

**TABLE 1:** Electrophysiological variables before (ΣM_MAX_), as well as at the beginning and the end (ΣRMS/ΣM_MAX_, CAR) of each MVC_1-MIN_ for 13 subjects (mean (standard deviation)).

		**Pre**		**Post**		**Post-10**	
		***Beginning***	***End***	***Beginning***	***End***	***Beginning***	***End***
ΣRMS/ΣM_MAX_ (10^-2^ mV)							
	**NMES 20**	3.0 (0.9)	1.8 (0.6)^*^	3.1 (0.9)	1.8 (0.5)^*^	3.0 (1.1)	2.3 (0.5)^$^
	**VOL 20**	2.9 (0.6)	1.9 (0.6)^*^	2.6 (0.8)	1.8 (0.7)^*^	2.8 (0.8)	2.3 (1.0)
	**NMES 40**	2.9 (0.9)	1.7 (0.5)^*^	2.6 (0.7)	1.4 (0.5)^*^	2.7 (0.7	2.0 (0.5)^*^
	**VOL 40**	2.8 (0.7)	1.6 (0.4)^*^	2.3 (0.6)^*^	1.4 (0.4)^*^	2.4 (0.7)^*^	1.9 (0.8)^$^
CAR (%)	**NMES 20**	98.6 (1.1)	93.7 (4.8)^*^	97.6 (1.5)	88.9 (11.0)^*^	97.6 (1.4)^‡^	91.9 (6.4)^*^
	**VOL 20**	98.4 (1.1)	95.5 (3.7)^*^	97.9 (1.5)	91.1 (10.9)^*^	97.2 (1.5)^‡^	92.0 (6.6)
	**NMES 40**	98.5 (0.9)	94.1 (3.8)^*^	98.1 (1.5)	87.5 (8.3)^*^	97.4 (1.4)^‡^	90.6 (7.6)^*^
	**VOL 40**	98.3 (1.1)	92.5 (5.4)^*^	94.9 (4.2)^‡^	83.2 (10.4)^*†^	95.7 (4.6)	88.3 (10.0)^*^

		**Pre**	**Post**		**Post-10**	
ΣM_MAX_ (mV)	**NMES 20**	22.6 (7.1)	21.9 (6.2)		21.5 (6.3)	
	**VOL 20**	24.5 (7.9)	23.8 (7.6)		22.1 (7.7)	
	**NMES 40**	25.0 (10.5)	19.1 (6.2)		20.1 (5.2)	
	**VOL 40**	25.8 (10.5)	24.6 (8.6)		24.5 (9.0)	

MVC = maximal voluntary contraction; MVC_1-MIN_ = sustained 1-min MVC; Pre and Post = before and after the fatiguing exercise respectively; Post-10 = after the 10-min rest period; VOL = voluntary fatiguing exercise; NMES = neuromuscular electrical stimulation fatiguing exercise; 20 and 40 represent the relative intensity of the fatiguing exercise (% of MVC). CAR = central activation ratio; ΣRMS = sum of electromyographic root mean square of triceps surae muscles; ΣM_MAX_ = triceps surae muscles sum of maximal M-wave.

* Different from the beginning value (*p* < 0.05).

† Different from the pre value (*p* < 0.05).

‡ Different from the pre value (*p* < 0.01).

$ Different from the post value (*p* < 0.05).

Before the exercise, no difference in ΣRMS_MVC_/ΣM_MAX_ was detected among sessions (Table 1; Z-range: 0.078–1.020; all *p* = 1.000; ES-range: 0.022–0.283). The ratio was reduced only by the VOL 40% MVC exercise (−17.2% ± 15.2%, Z = 2.830, *p* = 0.014, ES = 0.785), and was still depressed after the 10-min recovery period (−14.5% ± 15.0%, Z = 2.481, *p* = 0.039, ES = 0.688) (Table 1).

#### M_MAX_

The ΣM_MAX_ recorded at rest, before the fatiguing exercise, was similar among sessions, and was not modified neither by *time* and *condition* nor by the *intensity* (χ^2^ = 16.46, *p* = 0.125) (Table 1).

### Assessments related to maximal torque sustainability

#### Torque

**FIGURE 4:**
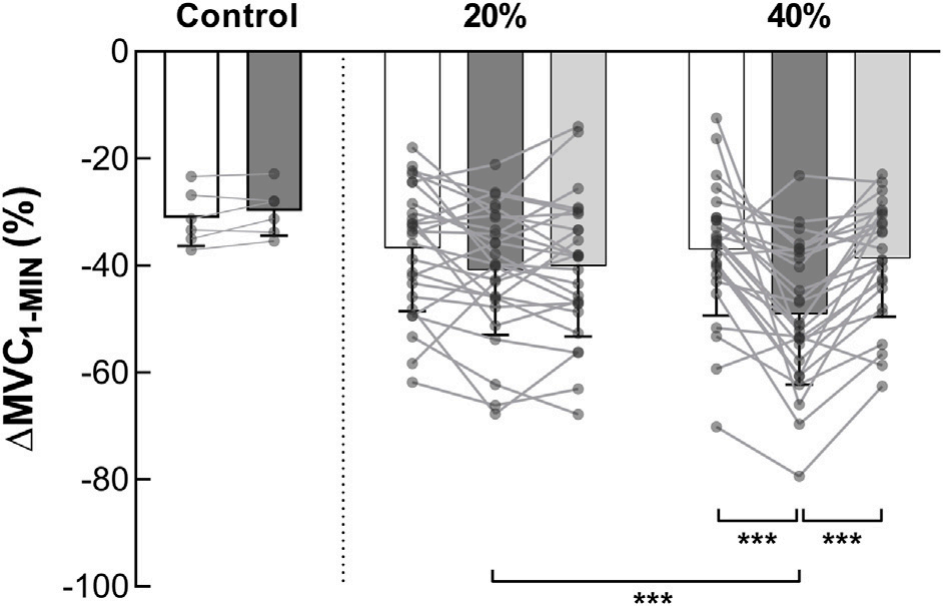
Torque loss during MVC_1-MIN_ (*n* = 13). ∆MVC_1-MIN_ represents the torque loss during the MVC_1-MIN_. Pooled ∆MVC_1-MIN_ data (NMES and VOL) at pre (white columns), post fatiguing exercise (dark grey columns) and post 10-min recovery period (light grey columns) are expressed as percentage of the pre-exercise MVC. Control represents the session without fatiguing exercise. *** Significant difference (*p* < 0.001). MVC = maximal voluntary contraction; MVC_1-MIN_ = 1-min sustained MVC.

A significant interaction effect (*intensity* × *time*) was detected on the ∆MVC_1-MIN_ (F_2,24_ = 8.988, *p* = 0.001, η_p_^2^ = 0.428) (Figure 4). Before the fatiguing exercises, no difference in ∆MVC_1-MIN_ was detected between the 20% and the 40% MVC sessions (*p* = 1.000, ES = 0.021). First, no change was observed following the 20% MVC exercises (pre = −36.72% ± 11.85% MVC vs. post = −40.86% ± 12.13% MVC, *p* = 0.196, ES = 0.345). Second, pooled data show that the decrease in torque during the 60-s MVC performed after the fatiguing exercise at 40% MVC (−49.10% ± 13.10%) was significantly greater than that observed before (−36.98% ± 12.36%, *p* < 0.001, ES = 0.952). In addition, the post 40% MVC exercise value was greater than the post 20% exercise one (*p* = 0.001, ES = 0.653). The 10 min of rest, after exercise at 40% MVC, allowed a complete recovery of the ∆MVC_1-MIN_ (Post-10 = −38.67% ± 10.90% MVC vs. pre, *p* = 0.920, ES = 0.198) (Figure 4). Since there was no change in ∆MVC_1-MIN_ after the 20% MVC exercises, no change was observed after the recovery period (−40.11% ± 13.11% MVC vs. pre, *p* = 0.389, ES = 0.271). Moreover, a positive correlation was detected (r_rm_ (38) = 0.383, *p* = 0.015, CI_95%_ = [0.072: 0.626]) between ∆MVC_1-MIN_ change and exercise TTI (Figure 3B).

#### CAR and RMS

**FIGURE 5:**
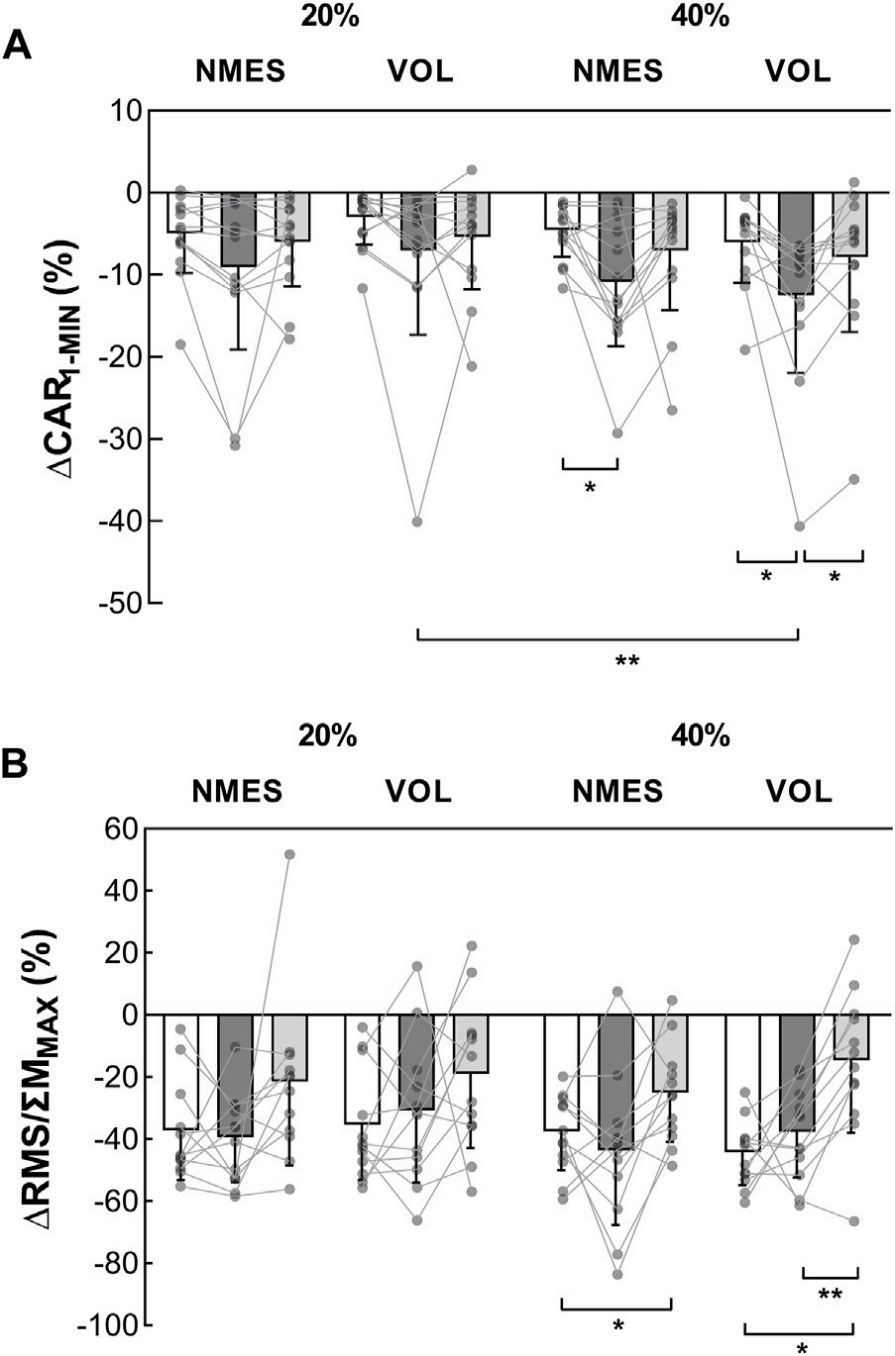
Evolution of voluntary activation indexes during the MVC_1-MIN_ (*n* = 13). **(A)** ∆CAR_1-MIN_ represents the reduction in CAR during the MVC_1-MIN_ (%). **(B)** ∆RMS/ΣM_MAX_ represents the evolution of the ΣRMS/ΣM_MAX_ during the MVC_1-MIN_ (%). These indexes were obtained before (white columns), after the fatiguing exercise (dark grey columns) and after the 10-min recovery period (light grey columns). *Significant difference (*p* < 0.05). **Significant difference (*p* < 0.01). MVC = maximal voluntary contraction; MVC_1-MIN_ = 1-min sustained MVC; CAR = central activation ratio; ΣRMS = sum of EMG root mean square of triceps surae muscles; ΣM_MAX_ = sum of triceps surae muscles maximal M-wave amplitude. VOL = voluntary fatiguing exercise; NMES = neuromuscular electrical stimulation fatiguing exercise; 20% and 40% MVC represent the intensity of the fatiguing exercise.

The ∆CAR_1-MIN_ was similar, before the fatiguing exercise, for all sessions (range: −2.98%—−6.01%; Z-range: 0.035–1.922; *p-*range: 0.328–1.000; ES-range: 0.010–0.533) (Figure 5A). Considering the fatiguing task, only the 40% MVC exercises had an impact on the ∆CAR_1-MIN_ (VOL: pre = −6.01% ± 4.96% vs. post = −12.46% ± 9.46%, Z = 2.551, *p* = 0.032, ES = 0.708; NMES: pre = −4.55% ± 3.29% vs. post = −10.86% ± 7.84%, Z = 2.760 *p* = 0.017, ES = 0.765). The 10-min recovery period was sufficient to restore the ∆CAR_1-MIN_ at its initial level after the 40% MVC exercises (Figure 5A). No difference in ∆CAR_1-MIN_ between pre and post-10 was observed for the exercises at 20% MVC.

Before the fatiguing exercise, no difference in ∆RMS/ΣM_MAX_ was detected among sessions (Figure 5B). Regardless of the session, the ∆RMS/ΣM_MAX_ was not altered by the fatiguing exercise. After the recovery, following the exercise at 40% MVC, the ∆RMS/ΣM_MAX_ was lower than before for both contraction modalities (NMES: pre = −37.6% ± 12.4% vs. post-10 = −25.0% ± 15.8%, Z = 2.510, *p* = 0.036, ES = 0.696; VOL: pre = −44.2% ± 10.4% vs. post-10 = −14.6% ± 23.3%, Z = 2.589, *p* = 0.029, ES = 0.718). No change was observed during the 20% MVC sessions.

## Discussion

The main finding of this study is that maximal torque production and sustainability (respectively quantified by MVC and ∆MVC_1-MIN_) are differently altered by fatiguing exercises consisting of intermittent voluntary and electrically induced contractions, performed at low and moderate intensities. Our results show that, unlike the MVC loss, ∆MVC_1-MIN_ is an index sensitive to the exercise TTI. Thus the association of these two indexes provide a more detailed description of the neuromuscular changes induced by a fatiguing exercise. In addition, the MVC_1-MIN_ has the further advantage of allowing the simultaneous assessment of both maximal torque production and sustainability. Additionally, it has been observed that the recovery of the maximal torque sustainability is faster than the recovery of the maximal torque production capacity.

### Maximal torque production

Since no difference in maximal torque production was observed between the brief MVC and the MVC_1-MIN_, performed before the fatiguing exercise, as well as an excellent reliability of this measure, it could be stated that the MVC_1-MIN_ provides a reliable index of the maximal torque production capacity, at least in an unfatigued muscle.

Our findings showed that, regardless of the contraction modality (i.e., NMES or VOL), MVC was reduced by the same amount after 20% and 40% MVC exercises and this despite the greater TTI for the higher contraction intensity. This finding is in line with the extensive literature comparing reductions in maximal torque production induced by exercises with different muscle TTIs, performed until exhaustion (Hunter et al., 2008; Rudroff et al., 2010; Baudry et al., 2011) or not (Vitry et al., 2019). Taken together, the present analysis and the foregoing studies seem to suggest that the exercise with the greatest muscle TTI is the exercise that surprisingly generates the smallest decrease in maximal torque production, as highlighted when the MVC loss is normalized by the TTI. A rational explanation for this is that MVC loss is not sensitive to exercise TTI, as evidenced by the absence of a significant correlation between these two parameters.

For the same contraction intensity, namely for a similar TTI, our findings show that VOL and NMES protocols induced an equivalent MVC loss. This result is in accordance with those of Doix et al. (2014) which show that NMES and VOL contractions of PF, at moderate intensity, brought about a similar MVC loss, when their TTIs were matched. However, it is noteworthy that most of the studies observed a larger MVC loss after NMES compared to VOL exercise, with an equivalent TTI (Theurel et al., 2007; Jubeau et al., 2008). This disagreement could be attributed to differences in the experimental paradigms of these earlier investigations compared to ours (e.g., frequency and intensity of electrical stimulation, duty cycle, target torque and the investigated muscles). Additionally, NMES stimulations used by Jubeau et al. (2008) and Theurel et al. (2007), delivered at the maximally tolerated intensity, induced a greater soreness compared with the submaximal VOL exercises performed in these same studies. Discomfort due to NMES may be responsible for the greater MVC loss despite the TTI being equivalent in both contraction modalities.

The contribution of nervous and muscular factors to the MVC loss could be investigated with several methods and techniques. One of these methods consists of using the central activation ratio as an index of muscle activation since it reflects the capacity to activate all the available motor units. In the current investigation, no effect of the contraction modality was observed on the investigated neural indexes (i.e., CAR and ΣRMS_MVC_/ΣM_MAX_ ratio) during the 20% MVC sessions, corroborating what was already observed by Doix et al. (2014). This indirectly suggests that the MVC loss could be mainly ascribed to muscular mechanisms. The absence of alteration in neuromuscular propagation (ΣM_MAX_), observed in the present study, suggests that changes in intracellular processes are implied. Such modifications that could have been assess by analysing the evoked twitches (Millet et al., 2012; Rozand et al., 2015) have not been done in the present study limiting in that way the direct assessment of alterations in muscle contractility.

On the other hand, at 40% MVC, a reduction in the CAR was observed, but only after the VOL exercise. Consequently, it seems likely that neural mechanisms contribute to the loss of MVC only after the VOL exercise at the higher intensity of contraction. This may, to some extent, be due to the fact that neural changes resulting from VOL exercise can be induced by both spinal and supraspinal mechanisms (Gandevia, 1998, 2001), while NMES, delivered at moderate intensity, seems to involve only supraspinal factors (Papaiordanidou et al., 2010; Alexandre et al., 2015).

As for the recovery, we observed that, whatever the fatiguing exercise, the maximal torque production capacity did not recover within 10-min rest. This observation is in accordance with data from several previous investigations showing that MVC was only partially restored after a rest period, even longer than 10 min (Jubeau et al., 2008; Chaubet et al., 2013). In our study, after the rest period, CAR values were still lower than pre-exercise, except for the VOL exercise at 40% MVC. However, in this session the CAR value at post-10 was not different from that post-exercise, and the latter was lower than that before the fatiguing exercise. Thus, it seems reasonable to presume that, overall, CAR did not recover after 10-min rest, this means that neural factors are responsible for the incomplete recovery of MVC.

### Maximal torque sustainability

The choice to set the duration of the sustained MVC to 1 min was made because the greatest torque loss generally occurs during the first minute of contraction (Gandevia et al., 1996; Kent-Braun, 1999; Post et al., 2009) Thus, this time frame makes the sustained MVC a useful index of maximal torque sustainability. The ∆MVC_1-MIN_ that we observed before the fatiguing exercise was about −37%, in accordance with the results of Moritani et al. (1985) which found a torque decline of −30%, in the same muscle group, after 60-s sustained contraction.

In order to investigate the nervous contribution to the torque loss during the MVC_1-MIN_, we analysed the CAR modification during this sustained contraction (∆CAR_1-MIN_). The ∆CAR_1-MIN_ was about −4% between the beginning and the end of the pre-exercise MVC_1-MIN_. Some studies showed that nervous alterations appear approximately 45 s after the onset of the MVC_1-MIN_ (Bigland-Ritchie et al., 1978; Schillings et al., 2003), explaining the small contribution of neural adjustments to the torque reduction throughout a maximal contraction of 1-min duration. The major part of the torque loss during the 1-min sustained contraction could, therefore, be accounted for by muscular changes. This result is in line with previous investigations which found that the greatest part of torque loss during a 1- to 3-min MVC is due to muscular alterations (Gandevia et al., 1996; Kent-Braun, 1999; Schillings et al., 2003; Place et al., 2007; Post et al., 2009; Vera-Ibáñez et al., 2018). In the present study, the maximal M-wave was not altered by the pre-exercise MVC_1-MIN_, as observed in previous studies for 45–120 s sustained MVC (Bigland-Ritchie et al., 1978; Place et al., 2007; Vera-Ibáñez et al., 2018). This finding indicates that the MVC loss should not depend on a change in neuromuscular transmission and propagation.

The current results showed that maximal torque sustainability was affected by the fatiguing exercise at 40% MVC, for both contraction modalities. Repeated measures correlation analysis confirms that the exercise TTI affects the neuromuscular sustainability, validating the hypothesis that ∆MVC_1-MIN_ is sensitive to muscle TTI, at least for submaximal exercises. This means, as expected, that the higher the TTI generated by the fatiguing exercise, the greater the torque loss during the sustained MVC. This also implies that the physiological mechanisms involved in protracting the MVC were more impaired after the exercise with the greatest TTI (i.e., 40% MVC). To better understand the underlying mechanisms, we examined changes in muscle activation during the sustained MVC. Our results showed that the greater ∆MVC_1-MIN,_ observed after the exercise at 40% MVC was associated with a greater ∆CAR_1-MIN_. Thus, the reduction in maximal torque sustainability may, at least in part, be attributed to a reduction in muscle activation capacity. This could be explained by the fact that the higher exercise intensity may cause a greater occlusion of blood vessels and thus reduce the muscle metabolite wash-out, compared with the lower intensity (Sejersted et al., 1984). The by-products accumulation during the fatiguing exercises could increase the stimulation of muscle afferents (in particular group III/IV muscle afferents) by receptors sensitives to mechanic and metabolic changes (Garland and Kaufman, 1995). These muscle afferents are known to have an inhibitory effect on the motor cortex, limiting the efferent motor command (Gandevia et al., 1996; Taylor et al., 2016). The lower intramuscular pressure induced by the 20% MVC contractions, compared with the 40% MVC contractions (Osada et al., 2015), leads to a lower accumulation of muscle metabolites, this could explain why the maximal torque sustainability was not altered by the fatiguing exercises performed at 20% MVC.

In addition, the greater muscle oxygen consumption, observed by Pethick et al. (2019) during an intermittent exercise at 40% MVC compared to 20% MVC, could be a further explanation for the different behaviour of the ∆MVC_1-MIN_ between these two intensities of exercise. Furthermore, the contribution of glycogen, as energy supply to produce adenosine triphosphate, increases with the intensity of the exercise (Holloszy et al., 1998). The glycogen is the main energy substrate between the sixth and the 50th second of a MVC_1-MIN_ (Lanza et al., 2005) and its depletion could partly account for the greater ∆MVC_1-MIN_ following the 40% MVC fatiguing exercises.

Concerning the contraction modalities, we observed a similar ∆MVC_1-MIN_ following the NMES and VOL exercises, whether the contraction intensity was 20% or 40% MVC. The reason could be that the muscle TTI was similar for the NMES and VOL exercises, at each contraction intensity. Hence, maximal torque sustainability seems to be mainly sensitive to muscle TTI than to contraction modality, at least when the fatiguing exercise is performed at low and moderate intensities.

We also investigated the recovery of maximal torque sustainability and we observed that the increased ∆MVC_1-MIN_, found after the exercise performed at 40% MVC (in both modalities), returned to baseline values after the rest period of 10 min. This suggests that this time frame is enough for a full recovery of maximal torque sustainability. The latter result allows us to refute the hypothesis according to which the exercises at 40% MVC induce a greater glycogen depletion. Indeed, the rapid and complete recovery of ∆MVC_1-MIN_ after the short rest period is inconsistent with the slow process of glycogen restoration. As observed by Robergs et al. (1991) it occurs hours for a complete glycogen renewal after an intermittent exercise at 35% MVC of the quadriceps muscle.

As previously mentioned, the partial occlusion of muscle blood flow, during the exercises at 40% MVC, may lead to the accumulation of by-products from metabolic processes reducing the central activation, with repercussions on the ∆MVC_1-MIN_. However, in a fatigued muscle, restoration of blood flow after circulatory occlusion attenuates the central inhibitory effect of group III/IV muscle afferent firing, which allows the recovery of voluntary activation capacity in ∼30 s (Kennedy et al., 2015). In our study, the ∆CAR_1-MIN_, after the 10-min rest period, returned to pre-exercise value for both VOL and NMES session at 40% MVC. This result supports the statement that the voluntary activation has a critical role in the maximal torque sustainability.

### Perspectives

The current study showed that MVC loss and ∆MVC_1-MIN_ have different evolution during various submaximal intermittent fatiguing exercises, as well as during a 10-min recovery period. Their divergent behaviour reveals that maximal torque sustainability, unlike maximal torque production, is sensitive to the exercise TTI. This highlights the importance to assess maximal torque sustainability, in addition to maximal torque production, in order to determine the neuromuscular impact of different fatiguing exercises. The MVC_1-MIN_, contrary to the brief MVC, has the advantage to provide simultaneously a reliable index of maximal torque production capacity and an assessment of maximal torque sustainability before and after the investigated muscle exercise. This protocol, using only a few peripheral nerve stimulations superimposed to the MVC_1-MIN_, is easy to set up in both healthy and pathological populations to monitor muscle training and deconditioning, and to discriminate neural and muscular contributions to torque production (Schillings et al., 2003). However, further investigations should be carried out to elucidate how these two physiological features (i.e., maximal torque production and sustainability) are modified as a result of different fatiguing protocols and recovery periods in main muscle groups. Finally, some computational models have been developed e.g., (Callahan et al., 2013, 2016; Eriksson et al., 2016; Herold et al., 2018; Rockenfeller et al., 2020) to predict the neuromuscular fatigability induced by different contraction conditions. Thus, it would be interesting to evaluate whether these predictive simulations fit with our experimental data. Also, it would be an interesting project in the future to see if such a model could predict the relation between exercise TTI and maximal torque sustainability we identified in the present study.

## Data Availability

The raw data supporting the conclusion of this article will be made available by the authors, without undue reservation.

## Abbreviations

∆CAR_1-MIN_, Change in CAR between during the MVC_1-MIN_; ∆MVC_1-MIN_, Torque change during the MVC_1-MIN_; ∆RMS/ΣM_MAX_, Change in muscle activity during the MVC_1-MIN_; ANOVA, Analysis of variance; CAR, Central activation ratio; EMG, Electromyographic activity; ES, Effect size; GL, Gastrocnemius lateralis; GM, Gastrocnemius medialis; ICC, Intraclass correlation coefficient; M_MAX_, Maximal M-wave; MVC, Maximal voluntary contraction; MVC_1-MIN_, 1-min maximal voluntary contraction; NMES, Neuromuscular electrical stimulation; PF, Plantar flexors; Rmcorr, repeated measures correlation; RMS, Root mean square; SOL, Soleus; TA, Tibialis anterior; TTI, Torque-time integral; VOL, Voluntary contraction; ΣM_MAX_, Sum of maximal M-wave of soleus and gastrocnemii; ΣRMS, Sum of EMG root mean square of soleus and gastrocnemii.
